# Pathophysiology, Management, and Therapeutics in Subarachnoid Hemorrhage and Delayed Cerebral Ischemia: An Overview

**DOI:** 10.3390/pathophysiology30030032

**Published:** 2023-09-14

**Authors:** Henry W. Sanicola, Caleb E. Stewart, Patrick Luther, Kevin Yabut, Bharat Guthikonda, J. Dedrick Jordan, J. Steven Alexander

**Affiliations:** 1 Department of Neurology, Louisiana State University Health Sciences Center in Shreveport, Shreveport, LA 71103, USA; dedrick.jordan@lsuhs.edu; 2 Department of Neurosurgery, Louisiana State University Health Sciences Center in Shreveport, Shreveport, LA 71103, USA; bharat.guthikonda@lsuhs.edu; 3 School of Medicine, Louisiana State University Health Sciences Center in Shreveport, Shreveport, LA 71103, USA; pml001@lsuhs.edu (P.L.); kgy001@lsuhs.edu (K.Y.); 4 Department of Molecular and Cellular Physiology, Louisiana State University Health Sciences Center in Shreveport, Shreveport, LA 71103, USA

**Keywords:** AHA, subarachnoid hemorrhage, cerebral vasospasm, delayed cerebral ischemia, aneurysm, intramural periarterial drainage, neuroinflammation, endothelial dysfunction, glymphatic system, pathophysiology

## Abstract

Subarachnoid hemorrhage (SAH) is a type of hemorrhagic stroke resulting from the rupture of an arterial vessel within the brain. Unlike other stroke types, SAH affects both young adults (mid-40s) and the geriatric population. Patients with SAH often experience significant neurological deficits, leading to a substantial societal burden in terms of lost potential years of life. This review provides a comprehensive overview of SAH, examining its development across different stages (early, intermediate, and late) and highlighting the pathophysiological and pathohistological processes specific to each phase. The clinical management of SAH is also explored, focusing on tailored treatments and interventions to address the unique pathological changes that occur during each stage. Additionally, the paper reviews current treatment modalities and pharmacological interventions based on the evolving guidelines provided by the American Heart Association (AHA). Recent advances in our understanding of SAH will facilitate clinicians’ improved management of SAH to reduce the incidence of delayed cerebral ischemia in patients.

## 1. Introduction

Subarachnoid hemorrhage (SAH) is defined as bleeding between the arachnoid and pia mater in the central nervous system (CNS). SAH is classified as either traumatic (an inciting event) or spontaneous (without an immediate identifiable cause). Traumatic SAH (tSAH) is bleeding into the subarachnoid space secondary to head trauma (e.g., falls, gunshot wounds, motor vehicle accidents) [1]. Spontaneous SAH (sSAH) is non-traumatic bleeding, most frequently caused by a ruptured cerebral aneurysm [2]. A further sub-classification of SAH is an aneurysmal SAH (aSAH) [3].

According to the most recent World Health Organization report, aSAH is 10-fold higher in Asia than in Europe [4,5,6]. Furthermore, low- to middle-income countries experience a disproportionately high burden (two-fold) compared to their higher-income counterparts [6]. These figures are disconcerting, as the incidence may be higher than reported since an estimated 12–15% of all aSAHs result in pre-hospital patient death [7,8].

Men have a higher incidence of aSAH between the ages of 25 and 45 or at older than 85 years of age, while women have a peak incidence of aSAH at 50–55 years of age [4,9,10]. Although the incidence of aSAH has decreased in women, they are still 1.24 times more likely than men to experience aSAH [8]. In fact, enhanced sex hormone-binding globulin and low bioavailable testosterone can enhance aSAH risk in women [10]. To further our understanding of the incidence and mechanism(s) of SAH, we have delineated the most important factors, such as inflammation, endothelial dysfunction, and blood within the subarachnoid spaces, followed by clinical implications and current treatment modalities.

## 2. Loss of Cerebral Autoregulation in SAH

The cerebral vasculature maintains blood flow through modulation of vascular tonicity [11]. This process, called cerebral autoregulation, ensures that perfusion is maintained over a broad range of cerebral perfusion pressures [12]. Cerebral autoregulation is primarily a non-neural, myogenic response to changes in transmural pressure (i.e., pressure across the arterial wall) [13,14]. Specifically, a physiologic increase in transmural pressure produces myogenic vasoconstriction, while a decrease in transmural pressure produces myogenic relaxation and vasodilation [15]. At the molecular level, cerebral autoregulation converts vascular wall tension into membrane depolarization and actin–myosin filament activity [16,17]. In contrast to cerebral autoregulation, neurovascular coupling is neural regulation of perfusion based on local metabolic activity [18]. These two different determinants of cerebral perfusion are often called “pressure” (i.e., cerebral autoregulation) versus “metabolic” (i.e., neurovascular coupling) autoregulation [13]. The function of cerebral autoregulation is the brain’s ability to maintain constant blood flow independent of changes in mean arterial pressure of 60–150 mm Hg [12,19]. Most interestingly, the loss of cerebral autoregulation has been associated with an increase in microvascular spasms [13], which have been correlated with the development of delayed cerebral ischemia (DCI).

A disastrous consequence of SAH is reduction in cerebral blood flow (CBF), which is often followed by DCI. Rupture of blood vessel(s) provokes a devastating cascade of events, which begin within minutes to hours after the initial insult and often lead to permanent disability or death [3]. Following uncontrolled elevations in blood flow, often reflecting high arterial blood pressure in the cranial vault, intracranial pressure (ICP) can quickly exceed physiological limits (ICP greater than 20–25 mm Hg) [20]. Blood pooling from the cranial vault and the expanding hematoma both contribute to elevated ICP, which can then herniate the brain, with life-threatening consequences [21].

The reduction in brain perfusion via aSAH is problematic, as brain tissue relies almost exclusively on aerobic respiration for support of its metabolism [22]. Even brief periods of hypoxia (loss of perfusion > 3–5 min) can lead to permanent neuronal tissue injury [23]. Initially, the hypoperfused tissue will upregulate anaerobic glycolysis, but this process cannot permanently sustain sufficient levels of adenosine triphosphate (ATP) [24], which is required for cellular homeostasis and energy-dependent processes (e.g., maintaining ionic gradients.) The loss of these ionic gradients can lead to cytotoxic edema and neuronal cell death [25].

## 3. Early Brain Injury and Acute Ischemia (0–3 Days)

Following the initial disruption to CBF via aSAH, blood enters the subarachnoid space and deposits fibrin, fibrinogen, and red blood cells (RBCs) [26]. Blood in the subarachnoid space rapidly disperses into the perivascular space (PVS), which is the area between the outer vascular wall and the glial limitans, which surrounds arterioles and capillaries and/or astrocytic feet [27]. Red blood cells are often lysed during SAH, resulting in elevated hemoglobin levels, which are then released into the brain parenchyma [28]. Since hemoglobin is primarily composed of ferritin, free iron from hemoglobin binds to tissues under oxygenation and generates free radicals, such as superoxide, nitric oxide and its metabolites, hydrogen peroxide, and oxidant heme through the Fenton reaction [29]. As a consequence, free radical production can enhance oxidative stress locally and in the brain penumbra [30,31,32,33,34]. Interestingly, deferoxamine (iron chelator) administered post-SAH can provide neuroprotection [31,33].

### 3.1. Glycocalyx

The glycocalyx is an important layer lining the internal wall of blood vessels as it can attenuate BBB permeability and is a vasculoprotective [35] barrier between the circulating blood and the endothelium facing the lumen [36,37]. The glycocalyx consists predominantly of proteoglycan and a glycoprotein backbone with glycosaminoglycan connections [35]. It is under constant remodeling due to hemodynamic changes, enzymatic degradation, and shear stress [36]. Interestingly, SAH can disrupt the glycocalyx, often associated with the upregulation of inflammatory cytokine production, reduced nitric oxide production, and neurovascular uncoupling [38].

aSAH can trigger upregulation of pro-inflammatory cytokines (e.g., TNF-alpha, IL-1, IL-6), which can in turn initiate cytokine-induced breakdown of the glycocalyx, leading to endothelium and adhesion molecule (i.e., VCAM, ICAM) dysfunction [38,39,40,41]. While glycocalyx degradation can expose the endothelium to pro-inflammatory cytokine-mediated damage, it also reduces endothelial nitric oxide [38].

During SAH, glycocalyx degradation occurs from the increased expression of inflammatory markers (e.g., IL-1, IL-6, TNF-alpha), atrial natriuretic peptides, and vascular shear stress [37]. Following disruption of the glycocalyx, endothelial NO production is disrupted, leading to vascular smooth muscle cell-mediated vasoconstriction [42]. Additionally, glycocalyx’s breakdown exposes its molecular backbone, which contains the molecules glypican and heparin sulfate, both of which serve to increase neutrophil migration and adhesion and potentiate platelet activation [43,44]. These compounding factors lead to further vascular compromise.

### 3.2. Endothelial Dysfunction and Neuroinflammation

Endothelial dysfunction in aSAH is characterized by the loss of NO production, unregulated coagulation (via coagulation cascade dysfunction), and enhanced permeability due to glycocalyx impairment [45,46]. Loss of the glycocalyx promotes coagulation since anticoagulant molecules (e.g., anti-thrombin, tissue factor inhibitor pathway, and vWF) are decreased to prevent platelet adhesion and aggregation [38]. aSAH-mediated damage to the endothelium can also decrease prostacyclin release, which prevents platelet adhesion [38]. Furthermore, TNF-α stimulates exposed endothelium to upregulate P-selectin and increase platelet–leukocyte–endothelial cell interactions [47,48].

### 3.3. Ischemia, Endothelial Dysfunction, and ACE-2: ‘Death in Rigor’

In our recent research, we discovered that ischemic stress to the cerebrum triggers a progressive, significant loss of perfusion, which occurs within the first 24 h, setting off a cascade of events that can have profound implications for patients’ outcomes [49]. Our investigations revealed that the progressive loss of perfusion is closely linked to the disruption of ACE-2-dependent vasodilation and anticoagulation mechanisms [50,51,52]. ACE-2, known for its vital role in regulating blood vessel tone and preventing excessive blood clotting, seems to be compromised under ischemic stress, as may occur during SAH, and post-ischemic vasoconstriction may reflect the constriction and death of microvascular pericytes and intense vasoconstriction, described by Hall et al. as ‘death in rigor’ [49]. Death in rigor may have adaptive value in very small regions of intense ischemia, in which it may control hemorrhage, but globally or regionally, such disruption leads to compromised perfusion, depriving large areas of the brain of essential oxygen and nutrients [53]. The consequences of reduced perfusion are devastating and may mediate injury in SAH, as has been found in ischemic stroke. Large regions of the cerebral volume become susceptible to ‘death in rigor’ [49] and may be sacrificed in the brain due to severe lack of blood flow, resulting in irreversible damage and impaired functionality. An improved understanding of the complex relationships among ischemic stress, ACE-2, perfusion loss, and ‘death in rigor’ may enable innovative approaches that can mitigate the consequences of both SAH and ischemic stroke [49].

### 3.4. Neuroinflammation

aSAH can lead to infiltration of neutrophils at the site of injury, along with systemic neutrophilia via enhanced IL-6 [54]. This process precipitates neutrophil recruitment to the brain between 12 and 48 h following aSAH [55]. Additionally, microglia activation and monocyte recruitment readily occur within the first 48 h of aSAH to promote neuroinflammation [55]. Neutrophil accumulation on endothelial membranes increases oxidative stress (via myeloperoxidase) and lipid peroxidation, resulting in endothelium damage [53,54,56,57]. Toll-like receptor 4 (TLR4) is enhanced in the activated microglia and macrophages, resulting in the secretion of TNF [58]. Free heme from RBC hemolysis forms reactive oxygen species, resulting in upregulation of metalloproteinase-9 (MMP-9) and breakdown of the vascular basement membrane [59].

### 3.5. Astrocytes

Naïve astrocyte activation occurs during SAH, transforming the cells into the A1 neurotoxic phenotype [60,61]. The A1 phenotype is characterized by upregulation of glial fibrillary acidic protein (GFAP), S100 calcium binding protein B (S100B), and C3, H2-D1, and serping1 cell markers [62]. A1 astrocytes are a form of reactive astrocyte, promoting cell death by releasing proinflammatory cytokines [61]. A2 astrocytes are another phenotypic variant in reactive astrocytes that play a neuroprotective role, in contrast to the dominant A1 neurotoxic phenotype found in SAH [63,64,65,66]. Reactive astrocytes (RAs) display a spectrum of pro- and anti-inflammatory profiles, rather than the simple A1 and A2 phenotypes based on genetic sequencing [67]. Overall, the pro-inflammatory RAs found in SAH exhibit strong neurotoxicity—forming fewer synapses and killing neurons with detached axons [68,69]. Activated astrocytes also undergo morphological changes after SAH, such as end feet swelling and protrusions compressing the capillary lumen [70].

### 3.6. Glymphatic System

**Figure 1:**
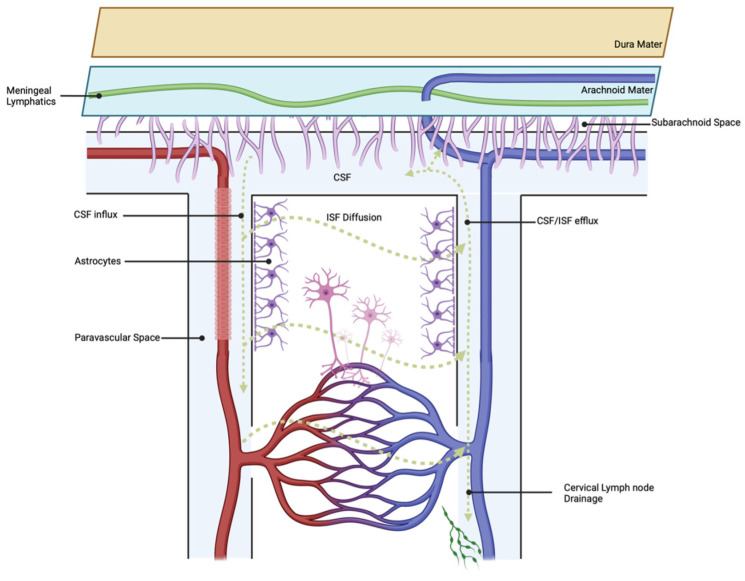
Glymphatic outflow proceeds via the mechanism described above, resulting in the clearance of toxic waste solutes under normal physiologic conditions.

The glymphatic system (GS) is a fluid exchange and drainage system supported by glial cells [71]. The GS includes the entire peripheral vascular system and consists of periarterial cerebrospinal fluid inlets and perivenous interstitial fluid (ISF) outlets [71]. Astrocytic end plates mediate the transport of metabolites via aquaporin-4 (AQP4) channels [72]. This transport system was confirmed with the use of fluorescent tracers administered into the brain parenchyma, which showed deposition in the meningeal lymphatic vessels and ultimately the deep cervical lymph nodes (dCLNs) [73]. Overall, the glymphatic system facilitates transport and clearance of metabolic waste under normal physiologic conditions [74], while during SAH, the glymphatic system is involved in the clearance of neurotoxic solutes, pro-inflammatory cytokines, and erythrocytes [71,75]. This process can be visualized in Figure 1 below.

After the initial SAH event, blood and its metabolites rapidly leak into the PVS. Blood in the PVS diffuses into the perivascular parenchyma via the GS, leading to a cascade of glial activation, neurotoxicity, and widespread microcirculatory dysfunction in the brain [76]. A recent study performed by Chen et al. found that meningeal lymphatics drained extravasated erythrocytes into the cerebral spinal fluid [75]. Following experimental ablation of meningeal lymphatics via the injection of Visudyne, there was a measurable decline in RBCs found in the cervical nymph nodes [75]. Additionally, following meningeal ablation, neuroinflammation and neurologic deficits were increased in a rodent model. Persistent malfunction of glymphatic and meningeal drainage was observed in an SAH mouse model by Pu et al. [77]. Decreased tracer was observed in the SAH mice compared to controls in meningeal lymphatic vessels and dcLNs [77], suggesting that SAH impairs the ability of meningeal lymphatic vessels to drain cellular debris, immune cells, and inflammatory mediators [78,79]. Additionally, glial cells lining the glymphatic regions become activated in SAH mice, as determined by increased levels of IL-1β, IL-6, and TNF-α expression [77]. This process results in AQP4 upregulation surrounding the arteries, while the AQP4 expression remains the same at the drainage sites around the veins [80]. Consequently, impaired PVS and ISF flow reduces metabolite and hemorrhagic clearance, leading to intermediate injury via immune cell accumulation [81], BBB dysfunction [82], neuronal apoptosis [77], vasculitis [76], cerebral edema [83], and acute hydrocephalus [84].

### 3.7. SAH and the Intramural Periarterial Drainage (IPAD)

**Figure 2:**
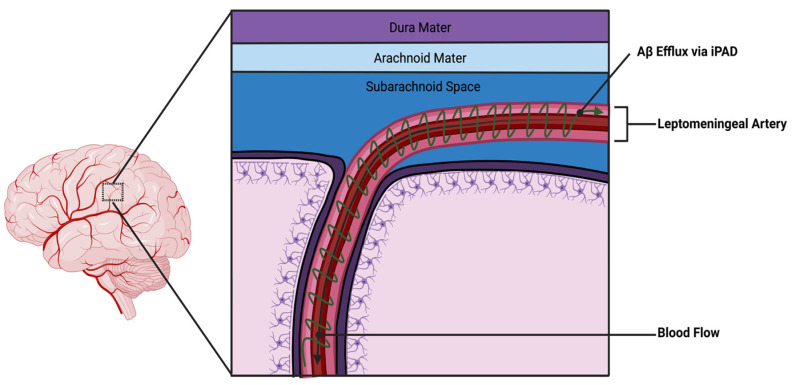
Pulsatile forces from the leptomeningeal artery drive retrograde CSF outflow along a periarterial path.

In addition to the glymphatic system, there is another drainage system called the intramural periarterial drainage (IPAD), which has been described as functioning in parallel with the glymphatic system. This system can also be affected during subarachnoid hemorrhage (SAH). Interstitial fluid (ISF), which is the fluid found between brain cells, can drain from the brain along the basement membranes of arteries through the IPAD pathway. Disruptions in the IPAD pathway can lead to problems with proteostasis in the brain, such as in cerebral amyloid angiopathy or Alzheimer’s disease. A study by Sun et al. [85] examined disturbances in the IPAD pathway after SAH in rats. In that study, the authors injected dyes of different sizes into the cisterna magna, a fluid-filled compartment at the back of the brain in the posterior cranial fossa. The cisterna magna is one of the subarachnoid spaces, which is filled with cerebrospinal fluid (CSF) and surrounds the brain and spinal cord. Sun’s group observed that these dyes entered the brain through periarterial channels and were cleared through the basement membranes of the brain’s associated capillaries. Interestingly, different molecular weight tracers showed different patterns of clearance. SAH significantly disrupted the IPAD pathway, causing enlargement of spaces around the blood vessels where ISF clearance was reduced. This effect was related to endothelial cell death, activation of astrocytes (a type of brain cell), increased levels of matrix metalloproteinase-9, and loss of type IV collagen in the basement membrane. Consequently, experimental SAH in rats has been found to significantly disrupt the IPAD pathway, which could have important clinical implications for SAH. These implications include problems with proteostasis, amyloid angiopathy, and other forms of abnormal protein accumulation in the brain associated with neurodegeneration. A representation of the IPAD mechanism can be seen in Figure 2 below.

## 4. Intermediate Injury (3–5 Days)

### 4.1. BBB Dysfunction

A hallmark of SAH is BBB degradation [86] in the form of endothelial and pericyte dysfunction [87]. Extracellular matrix proteins are also degraded following SAH-mediated degradation of endothelial cells [88].

Cytokines are locally released following SAH-mediated neuroinflammation to degrade cell adhesion molecules (i.e., ICAM-VCAM). Moreover, SAH-mediated neuroinflammation can cause a leaky BBB, which has been shown to decrease tight junction proteins such as ZO-1, occludin, and JAMa.

Pericytes are also important regulators in response to the change in cerebral blood flow with respect to local neuronal activity [89,90,91]. Pericytes also regulate and maintain the BBB by supporting tight junctional proteins [92]. Three to five days after brain injury, glycocalyx breakdown and endothelial dysfunction lead to increased BBB permeability [93]. BBB leakage allows for plasma protein extravasation, such as albumin, activating astrocytes, which in turn disrupt the neurovascular coupling [94]. More importantly, due to increased BBB leakage, extravasation of plasma proteins into the brain parenchyma is a major contributor to brain edema [94,95].

Pericytes amplify the inflammatory response by increasing their expression of microglial markers [96], upregulating periarterial AQP4 anchored in astrocytic end feet [97,98] and enabling extravasation of immune cells [99]. Smooth muscle cells (SMCs) undergo phenotypic transformation and migrate to the subepithelial layer [100]. IL-1β stimulates SMC proliferation in cerebral arteries and arterioles [101] and pericyte invasion of capillary networks, producing basement membrane remodeling [102]. Migration of transformed pericytes and SMCs increases the propensity for vessel spasm [103,104,105]. EC apoptosis starts around 24 h following SAH [106].

Astrocytes also modulate local vasoconstriction and vasodilation by releasing vasoactive compounds that stimulate 20-hydroxyeicosa-tetraenoic acid (20-HETE) production in pericytes [107], Intracellular Ca^2+^ concentrations in pericytes are also regulated by astrocytes promoting contraction [89,108]. Upregulation of AQP4 in astrocytes accelerates the formation of cytotoxic brain edema, leading to intracellular water accumulation [109,110]. Early in SAH, cellular edema in astrocytes produces an influx of ionic and vasogenic edema, leading to parenchymal edema [95]. Ionic edema develops when osmotic forces in the vasculature propel plasma through the vessel wall into the CNS [95]. The trans-endothelial Na^+^ gradient increases across the endothelium, leading to Na^+^ accumulation in the brain parenchyma [111]. This effect is magnified with early-onset CSF hypersecretion by the choroid plexi during SAH [112,113] and declining ISF efflux [114]. The large increase in parenchymal influx from extravasation and hydrocephalus exceeds the clearance rate provided by the glymphatic system. Tight junction breakdown and EC dysfunction contribute to vasogenic edema (e.g., extravasation of plasma proteins) [95]. Increased BBB permeability, cell death, and microvascular spasm and decreased ISF waste clearance escalate shifts in ion and water balance [95,115,116]. This process leads to neurovascular uncoupling, resulting in increased neuron susceptibility to terminal injury from cortical spreading depolarizations [117], excitotoxicity [118], neuronal apoptosis [81], and secondary brain injury [119].

### 4.2. Neurovascular Uncoupling

The neurovascular unit (NVU) is comprised of the BBB (e.g., ECs, pericytes, SMCs, astrocytes) and its communication with neurons and microglia [120]. The NVU serves to modulate pressure to local regions under normal physiologic conditions, such as regions that process reading, arithmetic, languages, etc. The NVU can also respond to pathophysiologic stimuli, such as seizures or CSD. The neurovascular unit is coupled when the local blood supply matches neuronal demand via modulation of vascular diameter [121]. The cellular interactions in the NVU dynamically regulate this activity [122]. Glutamate released from neurons stimulates nearby astrocytes and pericytes, generating vasoactive molecules [123]. The concentration of vasoconstrictor and vasodilator mediators determines the tone of the surrounding vasculature, modulating CBF [123]. In this model, neurons are “pacemakers” within the NVU model in that they regulate CBF [122]. For example, administration of catecholamines (e.g., dopamine, norepinephrine, and epinephrine) to neurons modifies EC tight junction protein production, thus changing BBB permeability [124]. All components in the NVU may modulate BBB maintenance [125]. Similarly, these components all coordinate to tightly control the CNS ionic microenvironment and ensure optimal neuronal functioning [126]. These processes include having specialized functions for neurotransmitters, maintaining low protein concentrations, preventing CNS exposure to neurotoxins, and limiting inflammatory processes [126]. The main function of astrocytes in the NVU is to regulate nutrient exchange by changing the tight junction density [127,128,129].

BBB breakdown following SAH disrupts the NVU, leading to neuronal hyperexcitability [130]. Neuroinflammation occurring in the CNS decreases the seizure threshold due to rapid changes in glutamate and γ-aminobutyric acid (GABA) receptor phosphorylation and channelopathies [131,132]. In rodent and pig models, BBB breakdown synchronized neuronal activity and increased seizure activity [133,134]. Albumin extravasation contributes to neuronal excitotoxicity by disrupting astrocytic K^+^ buffering capacity [130]. Albumin injection in naïve rats downregulated astrocytic Kir4.1 channels, leading to transient spiking activity [135]. These findings support neuroinflammation and albumin influx as drivers of neuronal network hyperexcitability [127].

During the intermediate phase (3–5 days post-SAH) neurovascular coupling is compromised following BBB dysfunction [136]. The brain tissue is particularly vulnerable during this period (3–5 days post-hemorrhage); cerebral vasospasm (CVS) is likely to occur during this period and further exacerbate damage to the brain parenchyma [137]. Neurovascular uncoupling occurs when there is a mismatch between blood supply and neuronal demand [38]. Uncoupling is exacerbated by neuronal hyperexcitability (e.g., increased metabolic activity), dysregulated vascular contractility, and decreased metabolic waste clearance, thus reducing the neuronal energy supply, leading to apoptosis [138]. The glymphatic system continues to deteriorate as SAH progresses, with loss of gap junctions (GJ) connecting neighboring astrocytes [139,140]. GJ loss limits neurotransmitter uptake, ion buffering, and glucose distribution for stable neuronal activity [141,142,143].

## 5. Delayed Injury (5–14 Days)

Loss of astrocytic GJs contributes to astroglial network compromise and subsequent astrocytic apoptosis [144,145]. However, loss of the astrocytic GJ loss diminishes the spread of Ca^2+^ waves, preventing synchronized activity between neurons and vascular SMCs [146]. Concurrently, disconnection between neurons and the supporting astroglial network hinders the neuronal energy supply [147]. In the setting of hypoglycemia or periods of neuronal hyperactivity, astrocytes monopolize the energy supply [148,149]. This cellular strategy provides another energy source (e.g., astrocyte–neuron lactate shuttle) for neurons during periods of excessive energy demand [150]. In SAH, astrocytic apoptosis is favored to precede neuronal apoptosis [145].

The deregulated neuronal activity of astrocytes depletes the neuronal energy supply and disrupts Na^+^–K^+^ pumps, which maintain ion homeostasis [151]. The means for neuronal ATP production are also impaired without the supporting astroglial network [151]. Injured neurons incur further injury from reactive Ca^2+^ influx, leading to large amounts of glutamate release, triggering local depolarizations [152]. Cortical spreading depolarizations (CSDs) consist of recurrent waves of neuronal and glial depolarizations that propagate widely from the onset zone [153]. CSDs begin under hypoxic conditions, strained energy supplies (e.g., glucose), and exposure to oxyhemoglobin following SAH [154,155]. CSDs peak at days 5–7 following aneurysmal SAH [156]. Changes in the neuronal microenvironment lead to massive glutamate release, loss of membrane potential, and CSD throughout the brain parenchyma [157]. If injured neurons cannot restore membrane potentials using Na–K^+^ pumps, the associated neurons and astrocytes swell and distort the dendritic connections [158]. CSDs eventually evolve into epileptiform discharges or pathologic changes in electrical potentials, silencing brain electrical activity [159]. Epileptiform discharges transmit greater disturbances in ion homeostasis between the intracellular and extracellular environments, resulting in neuronal swelling and glutamate receptor upregulation [159,160]. Underlying epileptiform discharges are neurons stuck in field potentials that are less than the inactivation threshold for channels generating action potentials [154]. Thus, neurons fire at high frequencies, producing moderately sustained depolarizations and less hyperpolarization [154,161].

### Cellular Changes

When metabolic demand exceeds the energy supply provided by the neurovascular unit (e.g., glucose delivery, cerebral blood flow), neuronal ischemia and apoptosis occur [162]. Within minutes, neuronal necrosis surpasses apoptosis as the primary form of cell death within the brain’s parenchyma [163]. The neuroinflammatory response is caused by an increase in neuroinflammatory signaling agents, such as IL-6, PAF, T-cells, and macrophages. The cytokines/chemokines involved are p53, Fas/FasL, tumor necrosis factor receptor, caspase 9 and apoptosis inducing factor [164]. These cytokines and chemokines lead to increased cell death of the brain’s parenchyma [165].

During the late or delayed stage (5–14 days post-SAH), CSDs peak in their occurrence. These peaks are associated with loss of neurovascular autoregulation. This loss is due to deranged NO/NOS signaling following changes to eNOS, nNOS, and iNOS [166]. During this phase, RBCs begin to break down/lyse within the subarachnoid spaces and increase oxyhemoglobin and ferritin concentrations within the brain parenchyma, causing further cerebral vasospasm [167].

The mass effect within the cranial vault physically displaces the brain parenchyma. The displaced parenchyma moves from a higher-pressure region (adjacent to the growing hematoma) to lower-pressure/resistance region(s) (e.g., lumens [openings] within the cranial vault). This process is problematic since, within the cranial vault, these openings are occupied by critical structures (e.g., brainstem, cranial nerves, vertebral arteries and veins) [168]. The brainstem controls functions such as respiration, blood pressure regulation, and other autonomic functions required for life. Compression of the brainstem leads to the loss of these critical autonomic functions [169,170,171,172].

Initially described by Ecker and Riemenschneider in 1951 [173], the association of CVS following SAH has been well documented. However, the exact mechanisms of CVS following SAH have been contested within the recent literature [174]. Previously, the accepted model of CVS, as described by Allcock and Drake [175], was that it occurred in the setting of focal ischemia experienced by the brain parenchyma during SAH and with a peak occurrence at 5–14 days post-initial hemorrhagic event [176]. Additionally, there exists a positive correlation between hemorrhage volume (HV) and the severity of CVS; this correlation has been recognized for its clinical utility and is used as the basis for the Fisher scale [177]. Recently, an ongoing clinical trial examined deferoxamine (i-Def), an iron chelating agent that sequesters the iron component of red blood cells. When i-Def was introduced into the brain’s parenchyma during a hemorrhagic event, initial results from second-phase clinical trials [178] were promising for reducing stroke sequalae [179]. If HV was less than 10 mL, the iatrogenic complications can outweigh any benefit that otherwise would be conferred by i-Def [180].

The link between cerebral vasospasm following the accumulation of blood degradation byproducts has been well established for several decades and was described by Toda and Ohta [181]. Recently, it has been shown that a potent inflammatory marker, oxyhemoglobin, is implicated in CVS, replacing the previously targeted methemoglobin and bilirubin as inflammatory markers [182,183]. The exact mechanism by which these substances induce CVS is still not well understood, although several features have been described. When injected intrathecally, oxyhemoglobin has been shown to induce CVS [174]. The mechanisms by which this outcome occurs is through the modification of normal expression of eicosanoids; oxyhemoglobin will increase the production of PGE_2_ and decrease PGI_2_ levels. Both eicosanoids are important in maintaining vascular tonicity. When oxyhemoglobin contacts methemoglobin, it spontaneously releases superoxide, which in turn causes lipid peroxidation, as well as vasoconstriction [174]. Additionally, this vasoconstriction is then compounded, as oxyhemoglobin has been shown to decrease the cerebral vasculature’s ability to relax [184]. Although there are ongoing studies to modify and alleviate these neuroinflammatory markers, a consensus has yet to be reached. This consensus has primarily been stymied by our partial understanding of the exact molecular pathways involved within the neuroinflammatory cascade. However, a reduction in the activity of the oxidative pathways [185], as described by the i-DEF trials, is promising in reducing neuronal damage in both the intermediate (within the first 3–5 days following SAH) and delayed (within the first 2 weeks) phases. Given that case fatality rates remain close to a third of patients suffering from intracerebral hemorrhage [186], early recognition and categorization by clinicians are imperative for treatment eligibility (e.g., i-Def), thereby minimizing cognitive and neurological deficits [187,188].

Additional substances released into the brain parenchyma during structural compromise of the vessel wall come from the spillage of intervascular wall components (intracellular enzymes and proteins) [189] that are cytotoxic to surrounding CNS tissue. Within minutes, there are gross anatomical and cellular changes to the CNS vasculature, e.g., mechanical/physical compression of neighboring/adjacent vessels, occluding their internal lumen(s), as well as compression of CNS tissue [190,191]. Regarding the cellular changes, notable is the disruption to chemosignaling agents (vasodilators/vasoconstrictors). These signaling agents under normal physiologic conditions are kept in a delicate balance to maintain the CNS vessels’ ability to autoregulate their tone [192].

Following the loss of cerebrovascular autoregulation, ICP equalizes to diastolic blood pressure (DBP) [191]. Given that DBP is several fold greater than normal CNS pressures (80–100 mm Hg and 7–15 mm Hg, respectively), the pressure differential can cause lateral displacement and subsequent compression of CNS tissue [193].

Ultimately, the compounding anatomical and cellular changes further contribute to a loss of micronutrients (perfusion) to local tissues and lack of clearance of toxic metabolic byproducts [191,194]. Within minutes of changes to perfusion, the cell’s ionic transmembrane gradient, which is crucial for normal neuro-signaling, becomes deranged [195]. This outcome follows the failure of adenosine triphosphatase’s ability to maintain the gradient in hypoxic conditions and thus its reliance on relatively ineffective anerobic pathways.

Following SAH, arterial vasospasm can occur, independently impairing perfusion (excluding non-spasmodic causes, e.g., clots, rupture of an atherosclerotic plaque, or embolus). Traditionally, this outcome has been detected by means of transcranial ultrasound (TCD) performed at the patient’s bedside approximately 2–3 days post-initial insult [196,197]. TCD is performed when vasospasm is suspected [198]. Currently, clinicians rely upon alterations in mentation or focal neurologic changes to escalate to imaging modalities for affected patients. In practice, these include shifts in the patient’s level of consciousness (neurobehavioral changes) or any new-onset focal neurologic defects [199]. However, this process can be challenging in patients who are intubated and/or comatose, especially for junior clinicians [200].

The recent literature has indicated that the link between vasospasm and DCI is tenuous, as the two have yet to be causally linked despite being the focus of many studies over several decades. These studies have utilized both clinical trials and animal models. Statistically, the evidence for a causal association is weak, as a DCI occurs 30–80% of the time following and/or during vasospasm. Additionally, CSD and DCI can occur without any antecedent (local or diffuse) vasospasm. Significant CVS (diffuse and/or involving several large caliber vessels) can occur without the presence of CSD [201].

**Figure 3:**
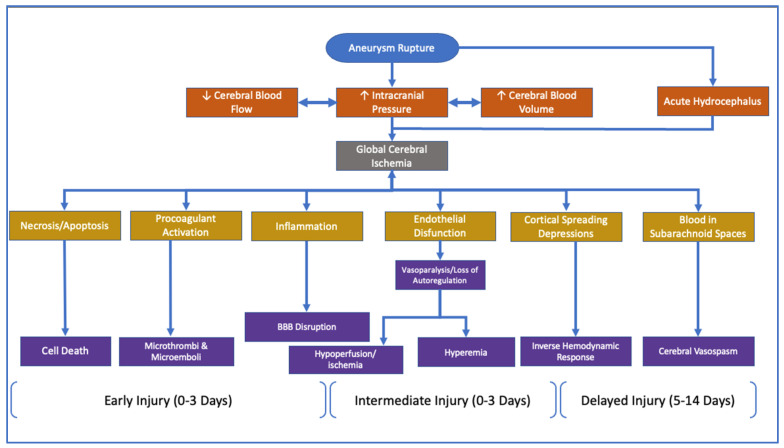
Chronological depiction of the proposed pathophysiological pathways for the development of DCI following SAH. Based on references: [163,165,208,209,210].

Historically, transcranial ultrasound has been used to diagnose the presence of CVS [196,197]. Transcranial ultrasound has the benefit of being a noninvasive, relatively inexpensive imaging modality compared to functional MRI. Although TCD can facilitate the activation of a CVS protocol, it is not without its shortcomings. Recent meta-analyses of clinical data have called into question the veracity of transcranial ultrasound as a reliable technique to diagnose the presence of CVS. Critics of this screening modality have cited the low specificity and widely variable sensitivity as the rationale for developing a new mainstay in the diagnosis of CVS [202,203,204]. Clinicians should consider CVS’s limitations; that is, it has higher sensitivity for larger caliber vessels that are more superficial. Clinicians also must consider that the spread of the vasospasm can be segmental in nature. The portion visualized by the TCD could appear as non-spasmodic, thus indicating a false negative, albeit while a distal portion of the vessel is in spasm [205]. Therefore, as an imaging modality, TCD is dependent primarily on the clinician’s experience and expertise to ensure that the entire vessel is swept in a longitudinal manner [196,206,207]. The timeline simplification of aneurysmal rupture, along with expected clinical signs and outcomes, can be seen in Figure 3 below.

## 6. Clinical Relevance and Management

aSAH patients present with a variety of symptoms, ranging from stiff neck, photophobia, loss of consciousness, and focal neurological deficits to altered mentation [211]. Approximately 80% of patients will state that they are experiencing “the worst headache of my life” [212]. Although prompt surgical intervention is required, preventing and treating SAH complications prior to surgery are tantamount. These presurgical interventions include the stratification of patients based on SAH severity (e.g., modified Fisher, Hunt Hess scales) and initiating anti-seizure therapy and nicardipine (infusions) for prevention of vasospasm [207].

Following aneurysmal rupture, a clinically observed sequence of events occurs. The presentation of SAH has been well known for decades due to its hallmark of patients reporting experiencing the “worst headache of my life,” clinically described as a “thunderclap headache” due to the rapid onset and the severity of the intensity. The vast majority of SAHs occur during nonstrenuous activities [213,214]. In addition to the thunderclap headache, more than two-thirds of patients will report nausea and/or vomiting, nuchal rigidity, altered level of consciousness, and/or a focal neurological defect. Especially worrisome is the occurrence of an antecedent (sentinel or warning) headache three months before the SAH, as these headaches are associated with a 10-fold increased risk of a rebleed following stabilization of the initial hemorrhagic event [215]. Sentinel or warning headaches are attributed to a minor hemorrhage prior to the major event. These symptoms can persist for several days [216,217] and merit consideration for the effectiveness of greater patient education for at-risk populations. That said, the continual challenge for clinicians is differentiating SAH’s sentinel headache from normal variants or those attributable to other causes because SAHs account for approximately 1% of all headaches presenting to emergency departments [218].

Reducing the time from symptom onset to neurosurgical intervention (i.e., coiling or clipping) is crucial in the reduction of morbidity and mortality [219]. Any patient whose presentation is suspicious for SAH should have radiographic imaging (non-contrast computer tomography or CT) performed. Following diagnosis of SAH, if a patient’s mental status deteriorates, current guidelines support the placement of an external ventricular drain (EVD) [207]. EVD placement is beneficial, as acute hydrocephalus is one of the most common complications in SAH (15–87% of SAH patients) [220]. EVD placement enables clinicians to monitor and reduce ICP via drainage of CSF. Additionally, placement of EVD in SAH-associated hydrocephalus is associated with neurological improvement [221,222,223,224]. EVD insertion serves to both continually monitor ICP and reduce intercompartmental swelling and pressure, thus improving cerebral perfusion pressure and metabolism [225]. If ICP remains elevated by more than 20 mm Hg, neurosurgeons may consider inserting a second EVD on the contralateral side or performing a decompressive craniectomy to reduce intracranial hypertension. Sedation (e.g., propofol, midazolam, fentanyl), osmotic agents (e.g., mannitol, hypertonic saline), barbiturate coma, and hypothermia may also be implemented systematically to lower ICP by 20 mm Hg before performing further surgical interventions [213,226]. In addition to ICP, brain tissue oxygen tension (PbtO_2_), delivery of oxygen (DO_2_), and jugular venous oxygen saturation (SjvO_2_) can also be monitored to optimize brain oxygenation and reduce metabolic stress [227]. The onset of brain hypoxia (PbtO_2_ < 20 mm Hg) initiates clinical interventions to optimize CPP, hemoglobin levels, intravascular volume, cardiac output, and oxygen saturation to minimize secondary brain injuries [227].

During the acute phase, clinicians optimize parameters to reduce the risk of rebleeding. Rebleeding is associated with a mortality rate as high as 70% and a poor prognosis for functional recovery. Computed tomography angiography (CTA) is performed to determine whether the patient has an aneurysmal subarachnoid hemorrhage. Digital subtraction angiography (DSA) remains the gold standard for detecting cerebral aneurysms.

Patients found to have an aneurysm undergo aggressive blood pressure monitoring and treatment to maintain systolic blood pressure less than 180 mm Hg per the European stroke organization guidelines and less than 160 mm Hg per the American Academy of Neurology guidelines [228]. Management of high blood pressure remains controversial due to the absence of any supporting clinical trials [229]. Data from observational studies suggest that aggressive management of hypertension can reduce rebleeding, but it is also associated with enhanced risk of secondary ischemia [3]. Another factor associated with rebleeding is an increased time between the rupture of the aneurysm and treatment.

Rebleeding occurs at a rate of 4–13.6% in the first 24 h, with maximal risk between 2 to 12 h post-aneurysm rupture [230,231,232,233]. An aneurysm not treated by endovascular embolization or neurosurgical clipping is considered unsecure, and intervention is performed as early as the patient is determined to be medically able, usually within 72 h after SAH. Postoperatively, obliteration of cerebral aneurysms is determined with a repeat DSA. Advantages and disadvantages exist for open versus endovascular treatments. Endovascular embolization is less invasive and was previously associated with a reduction in death and disability, fewer technical complications, reduced postoperative epilepsy, and less cognitive decline compared to clipping [234,235,236]. However, neurosurgical clipping is associated with a reduced incidence of late rebleeding (0.9% clipping vs 2.9% coiled) and a higher rate of aneurysm obliteration (81% clipped vs 58% coiled) [237]. Additionally, endovascular management is limited in its ability to obliterate small aneurysms (<3 mm) and is more costly than microsurgical clipping [238]. Given these differences, clipping is usually the preferred modality for middle cerebral artery aneurysms and patients with large intraparenchymal hematomas > 50 cc, but endovascular embolization is optimal for patients with a poor clinical grade, posterior cerebral artery aneurysms, or vasospasm or who are older in age [220].

Following stabilization of aneurysms, SAH patients are placed on neuromonitoring for vasospasm. This process requires hourly bedside neurological examinations and routine bedside TCD [239]. TCD is widely used as the primary clinical method for vasospasm screening. Detection of vasospasm by TCD activates therapeutic measures sooner and allows clinicians to monitor the patient’s response to treatment. TCD showing a mean flow velocity more than two standard deviations greater than the norm for individual intracranial vessels indicates vasospasm. However, the practitioners must be aware of TCD limitations, such as false positives (e.g., hyperemia, anemia) and false negatives (e.g., distal ACA regions), when interpreting the results [240]. Continuous electroencephalography (cEEG) can augment TCD assessments of vasospasm [241]. Quantification of relative alpha (RA) signals is associated with vasospasm [241]. Combining cEEG with neurological assessments and TCDs increases the sensitivity and specificity for vasospasm, along with surveillance for status epilepticus [242]. Acute neurological changes, in combination with vasospasm detected on TCD, are indications for CTA and CT perfusion (CTP). CTA and CTP are rapid, noninvasive modalities for visualizing vasculature, vasospasm, and the presence of infarction [243].

If symptomatic vasospasms do not respond to medical therapy, interventional cerebral angiography should be considered so that intraarterial therapy can be accomplished. During intraarterial therapy, several commonly used vasodilators, including verapamil, nicardipine, milrinone, and nitroglycerin, can be applied at the site of vasospasm to increase vessel diameters and downstream blood flow. However, if vasospasm remains unresponsive to pharmaceutical interventions, clinicians may escalate to balloon angioplasty (BA), which mechanically dilates the vessels. BA is superior in terms of therapeutic efficacy; however, there is a risk of arterial rupture and hemorrhage. Severe cases of vasospasm have received intrathecal injections of calcium channel blockers via EVD access. Intraventricular drug delivery has been effective in reducing the incidence of vasospasm and improving clinical outcomes [244]. Furthermore, there is not yet a consensus within the body of literature regarding the validity of other non-surgical interventions (triple-H therapy, high dose statins, and prophylactic calcium channel blockers). Triple-H therapy, or hypertension, hypervolemia, and hemodilution therapy, is used to maintain adequate brain perfusion in the setting of symptomatic vasospasm [245]. Randomized, controlled trials have argued against the use of prophylactic triple-H therapy for SAH patients on post-bleed days 0 and 14. Furthermore, there are no data from randomized trials showing improved outcomes with triple-H therapy despite its efficacy in augmenting CBF [246,247,248]. Intra-arterial interventions (e.g., angioplasty, verapamil) are the most effective treatments for large vessel vasospasm, while triple-H therapy is still utilized for mild vasospasm to avoid invasive procedures.

## 7. Conclusions

Although SAH accounts for a relatively small percentage (~15%) of strokes, those suffering from SAH are younger compared to the mean age of all stroke patients. This fact typically indicates a massive loss of life quality resulting from neurologic deficits experienced by these SAH patients.

The need for newer diagnostic procedures for SAH and the prevention of DCI is imperative, as the limitations of traditional methods (e.g., TCD) have become increasingly apparent. Cerebral vasospasm has proven to be an elusive phenomenon, both in detection and in its prognostic value. Examination of emerging data suggests a shift in focus to alternative detection and treatment modalities. Oxyhemoglobin is the new emerging biomarker implicated in the detection and intervention of CSD, replacing former markers. The exact molecular mechanisms by which oxyhemoglobin exacerbates DCI are currently unknown, but ongoing clinical trials have shown the benefits of curbing its presence. Early surgical intervention is a priority following stabilization of the patient. New findings from ongoing clinical trials merit the inclusion of non-surgical interventions during these early stages. These medical interventions are promising, such as the i-DEF trials.

Last, further appreciation of the contributing factors, as well as the exact mechanisms for reducing neuroinflammation, is crucial, as it plays a significant role in the migration of pericytes and smooth muscle to injured regions. Decreasing inflammation will decrease the migration of pericytes and subsequent DCI.

## Data Availability

Not applicable.

## Abbreviations

AQP4	aquaporin 4
CBV	cerebral blood volume
CSD	cortical spreading depressions
CTA	computed tomography angiography
CVS	cerebral vasospasm
DCI	delayed cerebral ischemia
EVD	external ventricular drain
GJ	gap junction
GS	glymphatic system
ICP	intracranial pressure
NVU	neurovascular unit
PVS	perivascular space
SAH	subarachnoid hemorrhage
aSAH	aneurysmal subarachnoid hemorrhage
sSAH	spontaneous subarachnoid hemorrhage
tSAH:	traumatic subarachnoid hemorrhage
TCD	transcranial Doppler (ultrasound)
